# Supplementary material for: Associations between Depressive Symptoms and Satisfaction with Meaningful Activities in Community-Dwelling Japanese Older Adults
Source: J Clin Med. 2020 Mar 14;9(3):795. doi: 10.3390/jcm9030795 (PMC7141267; doi:10.3390/jcm9030795)
Supplement: Supplementary file 1 [file jcm-09-00795-s001.pdf]

Supplementary Table S1. Proportions of sub-items in each meaningful activity categories.

	All participants (n = 806)	Non-depressive group (n = 679)	Depressive group (n = 127)
Self-care, n	90	74	16
Eating/drinking, n (%)	41(45.6)	33(44.6)	8(50.0)
Dressing, n (%)	1(1.1)	1(1.4)	0
Washing/bathing, n (%)	1(1.1)	1(1.4)	0
Maintaining one's health, n (%)	47(52.2)	39(52.7)	8(50.0)
Mobility, n	9	7	2
Driving, n (%)	8(88.9)	6(85.7)	2(100)
Using transportation, n (%)	1(11.1)	1(14.3)	0
Domestic life, n	144	112	32
Shopping, n (%)	4(2.8)	3(2.7)	1(3.1)
Cooking meals, n (%)	82(56.9)	67(59.8)	15(46.9)
Laundry, n (%)	8(5.6)	7(6.3)	1(3.1)
Cleaning, n (%)	7(4.9)	6(5.4)	1(3.1)
Household maintenance, n (%)	4(2.8)	4(3.6)	0

Child care, n (%)	11(7.6)	7(6.3)	4(12.5)
Assisting old people/patients, n (%)	12(8.3)	5(4.5)	7(21.9)
Collecting information, n (%)	10(6.9)	8(7.1)	2(6.3)
Makeup, n (%)	1(0.7)	1(0.9)	0
Management of property, n (%)	5(3.5)	4(3.6)	1(3.1)
Work/Education, n	55	49	6
Remunerative employment, n (%)	42(76.4)	37(75.5)	5(83.3)
Non-remunerative employment, n (%)	11(20.0)	10(20.4)	1(16.7)
Informal education, n (%)	2(3.6)	2(4.1)	0
Interpersonal interactions, n	154	134	20
Family relationships, n (%)	98(63.6)	88(65.7)	10(50.0)
Friendship, n (%)	53(34.4)	43(32.1)	10(50.0)
Formal relationships, n (%)	2(1.3)	2(1.5)	0
Imitate relationships, n (%)	1(0.6)	1(0.7)	0
Social life, n	77	65	12
Religion, n (%)	62(80.5)	50(76.9)	12(100)
Political life and citizenship, n (%)	1(1.3)	1(1.5)	0

Ceremonial functions, n (%)	1(1.3)	1(1.5)	0
Banquet/meeting, n (%)	1(1.3)	1(1.5)	0
Community activities (e.g. Parent-Teacher-Association), n (%)	6(7.9)	6(9.2)	0
Community events (e.g. festival), n (%)	6(7.9)	6(9.2)	0
Sport, n	87	80	7
Baseball/catch, n (%)	1(1.1)	0	1(14.3)
Ping-pong, n (%)	1(1.1)	1(1.3)	0
Martial arts, n (%)	1(1.1)	1(1.3)	0
Croquet, n (%)	29(33.3)	29(36.3)	0
Swimming, n (%)	1(1.1)	1(1.3)	0
Hiking, n (%)	2(2.3)	2(2.5)	0
Cycling, n (%)	1(1.1)	1(1.3)	0
Jogging/marathon, n (%)	1(1.1)	1(1.3)	0
Walking, n (%)	34(39.1)	31(38.8)	3(42.9)
Golf, n (%)	12(13.8)	9(11.3)	3(42.9)
Dancing, n (%)	4(4.6)	4(5.0)	0

Leisure, n	190	158	32
Painting, n (%)	4(2.1)	3(1.9)	1(3.1)
Reading, n (%)	10(5.3)	8(5.1)	2(6.3)
Handicrafts, n (%)	13(6.8)	12(7.6)	1(3.1)
Poetry, n (%)	1(0.5)	1(0.6)	0
Calligraphy/Flower arrangement/ Tea ceremony, n (%)	1(0.5)	1(0.6)	0
Making sweets, n (%)	2(1.1)	2(1.3)	0
Playing instruments, n (%)	3(1.6)	3(1.9)	0
Karaoke, n (%)	2(1.1)	2(1.3)	0
Horticulture, n (%)	78(41.1)	66(41.8)	12(37.5)
Do-it-yourself, n (%)	5(2.6)	4(2.5)	1(3.1)
Gambling, n (%)	1(0.5)	1(0.6)	0
Pet care, n (%)	15(7.9)	13(8.2)	2(6.3)
Diary/blog, n (%)	3(1.6)	2(1.3)	1(3.1)
Computer, n (%)	4(2.1)	3(1.9)	1(3.1)
Photography/Videography, n (%)	3(1.6)	3(1.9)	0

Traveling, n (%)	6(3.2)	5(3.2)	1(3.1)
Watching sport, n (%)	3(1.6)	2(1.3)	1(3.1)
Eating out, n (%)	3(1.6)	3(1.9)	0
Driving, n (%)	4(2.1)	4(2.5)	0
TV/radio, n (%)	13(6.8)	6(3.8)	7(21.9)
Fishing, n (%)	11(5.8)	10(6.3)	1(3.1)
Japanese dance, n (%)	4(2.1)	3(1.9)	1(3.1)
Music/movies at home, n (%)	1(0.5)	1(0.6)	0
